# Functional haemodynamic monitoring: the relative merits of SVV, SPV and PPV as measured by the LiDCOrapid in predicting fluid responsiveness in high-risk surgical patients

**DOI:** 10.1186/cc9486

**Published:** 2011-03-11

**Authors:** C Willars, A Dada, D Green

**Affiliations:** 1 Kings College Hospital, London, UK

## Introduction

Standard anaesthetic practice in the high-risk surgical patient is to insert invasive arterial and central venous catheters and then to use ΔCVP and ΔMAP to guide fluid therapy, despite an accumulation of evidence to suggest that filling pressures are inadequate predictors of fluid status and responsiveness. Recent interest has been directed towards dynamic measures of cardiac filling such as SVV, SPV, PPV and Δdown and ΔVpeak. A number of large multicentre trials are underway using the LiDCOrapid. There is, however, little information about the utility of this device or, indeed, any other minimally-invasive cardiac output monitor in the prediction of fluid responsiveness.

## Methods

The haemodynamic parameters of 70 high-risk patients (mean age 71 ± 11.3, median ASA 3) undergoing major vascular surgery (mean duration 4.2 ± 1.1 hours) were evaluated retrospectively using LiDCOviewPro. All patients underwent standard induction and maintenance of anaesthesia, with propofol/remifentanil TIVA and IPPV (tidal volume ≥7 ml/kg) via a supraglottic airway. Monitoring included BIS, NICO and LiDCOrapid. Fluids were administered according to clinical assessment of need and available haemodynamic parameters. Only fluid boluses given in the absence of HRV >10%, brisk ongoing blood loss and of volume ≥250 ml were included in the evaluation. Positive response to a fluid challenge was defined as ΔSVI ≥10%. Statistical analysis was performed using SPSS 17.0.

## Results

Thirty-two out of 43 valid fluid challenges were positive (74.4%). The correlation coefficients between the baseline SVV, SPV and PPV with ΔSVI were 0.27 (*P *= 0.08), -0.01 and 0.18 (nonsignificant). The AUROCs were 0.75 (95% CI = 0.57 to 0.93), 0.587 (0.36 to 0.82) and 0.67 (0.48 to 0.86), respectively. The best cut-off value for SVV using Youden's index was 13.5%, with *J *= 0.48. The positive likelihood ratio was 2.74 and the negative likelihood ratio 0.34, with diagnostic odds ratio 8.06 at this level.

## Conclusions

It has been reported that only 50% of critically unwell patients respond to fluid challenge, compared with 74.4% in this intraoperative study of noncardiac surgical patients. The SVV was an adequate predictor of fluid responsiveness. The diagnostic threshold of 13.5% was consistent with previous studies.

